# Probing the Field-Effect Transistor with Monolayer MoS_2_ Prepared by APCVD

**DOI:** 10.3390/nano9091209

**Published:** 2019-08-27

**Authors:** Tao Han, Hongxia Liu, Shulong Wang, Shupeng Chen, Haiwu Xie, Kun Yang

**Affiliations:** Key Laboratory for Wide-Band Gap Semiconductor Materials and Devices of Education, The School of Microelectronics, Xidian University, Xi’an 710071, China

**Keywords:** monolayer MoS_2_, FET, mobility, Raman spectrum, photoluminescence (PL) spectrum

## Abstract

The two-dimensional materials can be used as the channel material of transistor, which can further decrease the size of transistor. In this paper, the molybdenum disulfide (MoS_2_) is grown on the SiO_2_/Si substrate by atmospheric pressure chemical vapor deposition (APCVD), and the MoS_2_ is systematically characterized by the high-resolution optical microscopy, Raman spectroscopy, photoluminescence spectroscopy, and the field emission scanning electron microscopy, which can confirm that the MoS_2_ is a monolayer. Then, the monolayer MoS_2_ is selected as the channel material to complete the fabrication process of the back-gate field effect transistor (FET). Finally, the electrical characteristics of the monolayer MoS_2_-based FET are tested to obtain the electrical performance. The switching ratio is 10^3^, the field effect mobility is about 0.86 cm^2^/Vs, the saturation current is 2.75 × 10^−7^ A/μm, and the lowest gate leakage current is 10^−12^ A. Besides, the monolayer MoS_2_ can form the ohmic contact with the Ti/Au metal electrode. Therefore, the electrical performances of monolayer MoS_2_-based FET are relatively poor, which requires the further optimization of the monolayer MoS_2_ growth process. Meanwhile, it can provide the guidance for the application of monolayer MoS_2_-based FETs in the future low-power optoelectronic integrated circuits.

## 1. Introduction

The field effect transistors (FETs) are the basic unit of very large scale integrated circuits [1,2]. The feature size of the transistor has reached the physical limit with the integration of integrated circuits increases. Therefore, it is necessary to find the suitable semiconductor materials to improve the electrical performance of transistor [3,4]. Many researchers have focused on the two-dimensional materials with the single layer of atomic thickness [5]. The two-dimensional material is used as the channel material compared to the traditional bulk material, which can not only help to suppress the short channel effect, but also effectively reduces the static leakage current [6,7]. In addition, the two-dimensional materials also have the higher specific surface area, excellent mechanical strength, higher optical transparency, and various excellent photoelectric characteristics, so it can be widely used in the gas sensors [8], flexible electronics [9], and photodetectors [10]. Compared to the Si material, there are no dangling bonds in the low-dimensional transition metal sulfur compound materials when the transistor size is at the zoom limit [11]. The molybdenum disulfide (MoS_2_) has the semiconductor characteristics, excellent physical and chemical properties, and unique microstructure, which can directly construct the field effect transistor. Therefore, the MoS_2_ has become the very promising channel material in the process of the transistor scale.

As we all know, the thickness of the MoS_2_ sample obtained by the mechanical peeling is larger, and the MoS_2_ sample is smaller and irregular, so the MoS_2_ is grown on the SiO_2_/Si substrate by the atmospheric pressure chemical vapor deposition (APCVD) [12]. There are many factors that affect the continuity and uniformity of the MoS_2_ deposition while using the APCVD method to obtain the MoS_2_, such as the growth temperature, growth time, the amount of S powder and MoO_3_ powder, and the gas flow rate. The band gap of MoS_2_ changes with the number of layers, the bulk MoS_2_ has the moderate electron mobility and an indirect band gap of 1.29 eV, whereas the monolayer MoS_2_ is a direct bandgap material with a band gap of 1.8 eV. The MoS_2_ is a promising material for the flexible and transparent substrates, which can be applied in the logic circuits and optoelectronic devices [13]. The size and quality of MoS_2_ have a great influence on the performance of the device. Therefore, we can improve the continuity and uniformity of the MoS_2_ deposition by adjusting the growth process parameters and treating the SiO_2_/Si substrate with the graphene quantum dot solution [14]. The FETs with the direct bandgap monolayer MoS_2_ have the larger switching ratio and the lower off-state current. However, the mobility and on-state current of the FETs are very lower, so it is very meaningful to optimize and enhance the electrical performance, which can provide the application reference of the monolayer MoS_2_-based FETs.

The paper is composed of five parts: First, the large-area high-quality monolayer MoS_2_ is prepared by the APCVD to facilitate the fabrication of FETs [15]. Then, the monolayer MoS_2_ is confirmed and characterized by the high resolution microscopy, Raman spectroscopy, photoluminescence spectroscopy, and field emission scanning electron microscopy. Next, the main preparation process of monolayer MoS_2_-based FET is described. Subsequently, the electrical performance of the back-gate FET is measured. Finally, the conclusion of this paper is summarized. The following are the electrical performance parameters of the prepared monolayer MoS_2_-based FET in this paper, the electrical performance parameters have increased by improving the electrode contact and channel material [16]. The switching ratio is as high as 10^3^, the field effect mobility is about 0.86 cm^2^/Vs, the saturation current is 2.75 × 10^−7^ A/μm, and the lowest gate leakage current is 10^−12^ A. Besides, the monolayer MoS_2_ can form the ohmic contact with the Ti/Au electrode. Although the electrical performance of monolayer MoS_2_-based FETs is not ideal, we have mastered the fabrication process of monolayer MoS_2_-based FETs, and the growth process of monolayer MoS_2_ needs further optimization, which can provide the reference for the preparation of high quality monolayer MoS_2_-based FETs [17].

## 2. The Growth and Characterization of Monolayer MoS_2_

### 2.1. The Growth Process of Monolayer MoS_2_

In this paper, the monolayer MoS_2_ on SiO_2_/Si substrate was grown by APCVD. First, the solid sulfur powder and MoO_3_ powder could be melted into the gas state under the high temperature. Then the argon gas with a purity of 99.999% was passed as the carrier gas, and the sulfur gas was transferred to the vicinity of the SiO_2_/Si substrate. At the same time, there was a certain concentration of MoO_3_ vapor in the vicinity of the SiO_2_/Si substrate. Finally, the sulfur gas could react with MoO_3_ gas on the surface of the SiO_2_/Si substrate to form the MoS_2_. The specific growth experiment process of monolayer MoS_2_ was as follows: The SiO_2_/Si substrate was selected as the growth substrate of MoS_2_, wherein 300 nm SiO_2_ was the back gate dielectric layer of FET [18]. Before the growth experiment of MoS_2_, the 1 cm × 1 cm SiO_2_/Si substrate was first subjected to the oxygen plasma treatment. The vacuum tube furnace of the MoS_2_ growth experiment was TF55035C-1, and the front end is equipped with a heater that could be heated to 400 °C, which could help to assist the evaporation of sulfur powder. First, the quartz boat with 100 ± 5 mg sulfur powder (Alfa Aesar, Shanghai, China, 99.5%) was placed in the middle zone of the heater. Then, the quartz boat with 2 ± 0.1 mg MoO_3_ powder (Alfa Aesar, Shanghai, China, 99.95%) and SiO_2_/Si substrate was placed in the middle of the tube furnace. Next, the argon gas with 200 sccm was introduced into the tube furnace for 10 min to eliminate the air of the tube furnace. Subsequently, the temperature of the sulfur powder was heated to 200 °C, and the MoO_3_ powder was heated to 750 °C. During the growth of MoS_2_, the argon gas with a flow rate of 40 sccm was continuously provided and the growth temperature was maintained for 10 min [19]. Finally, the growth reaction of MoS_2_ was completed, and the MoS_2_ sample was taken out while the temperature of the tube furnace was cooled to room temperature.

### 2.2. The Test Characterization Conditions of Monolayer MoS_2_

The MoS_2_ sample could be obtained by the APCVD. At the same time, the MoS_2_ could be systematically characterized by optical microscopy, Raman spectroscopy, photoluminescence spectroscopy, and field emission scanning electron microscopy to further determine the layer number and quality of the MoS_2_ sample. The Raman model was LabRam HR Evolution with a laser wavelength of 532 nm (HORIBA JobinYvon, Paris, France) [20]. The specific test conditions of the Raman spectrometer were the 100× objective lens, 1800 groove/mm grating, the spot size of 532 nm laser was 342 nm, and the incident laser power density was 140 μW/μm^2^. Besides, the field emission scanning electron microscopy (FESEM, JSM-6700F, Hitachi, Tokyo, Japan) was also used at the accelerating voltage of 5 kV.

**Figure 1:**
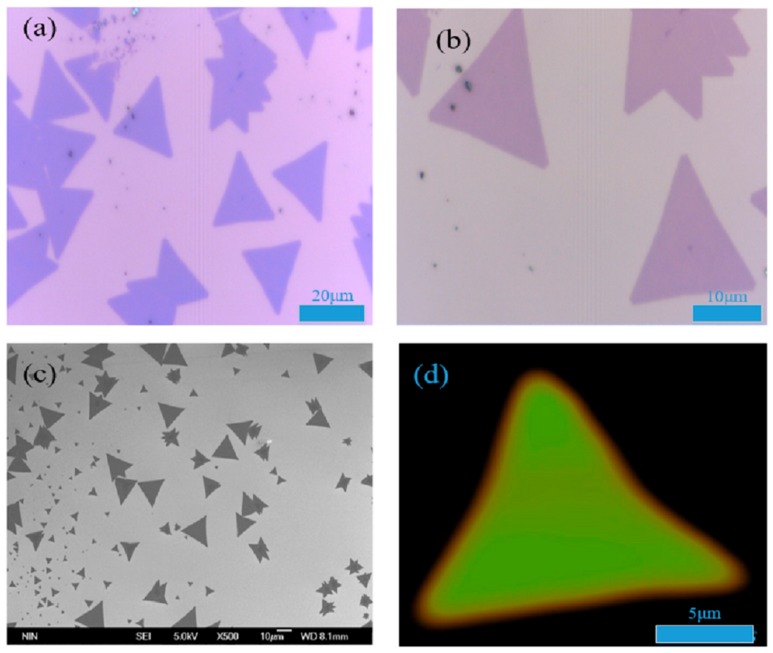
(**a**) The 50× objective optical micrograph of monolayer MoS_2_; (**b**) the 100× objective optical micrograph of monolayer MoS_2_; (**c**) the FESEM image of triangular monolayer MoS_2_; and (**d**) the mapping diagram of monolayer MoS_2_.

The monolayer MoS_2_ and the SiO_2_ of SiO_2_/Si substrate could interfere with the light, so there was a reflection enhancement effect on the visible light wavelength of 532 nm. Figure 1a,b respectively show the 50× and 100× objective optical micrograph of monolayer MoS_2_, the pink and blue patches in the optical images respectively represents the monolayer MoS_2_ on SiO_2_/Si substrate and the scale, it could be observed by the optical microscope that monolayer MoS_2_ on SiO_2_/Si substrate exhibited the bright blue color. As shown in Figure 1c, the typical FESEM image of monolayer MoS_2_ clearly exhibited the quasi-equilateral triangles, which was consistent with the crystal structure. Besides, the Raman mapping was tested to observe the film formation quality and uniformity of the triangular MoS_2_. It can be seen from the Figure 1d that the color of MoS_2_ mapping was relatively uniform, which could indicate that the sample was the high-quality uniform monolayer MoS_2_.

**Figure 2:**
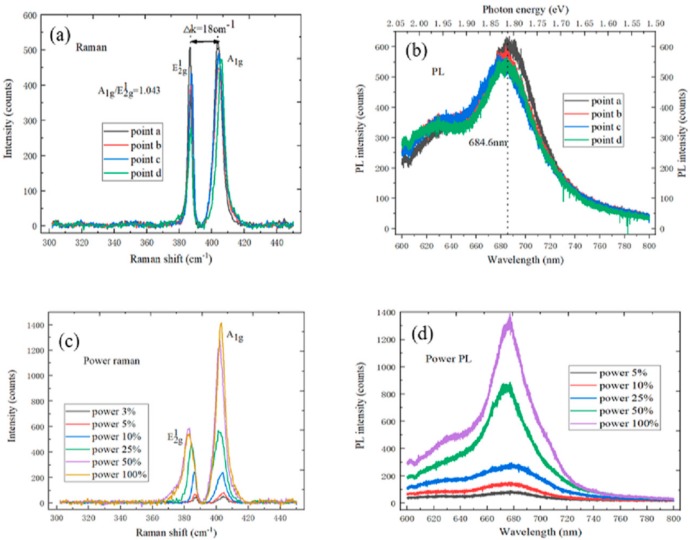
(**a**) The Raman spectrum of monolayer MoS_2_ at the different test points; (**b**) the PL spectrum of monolayer MoS_2_ at the different test points; (**c**) the Power Raman spectrum of monolayer MoS_2_; and (**d**) the Power PL spectrum of monolayer MoS_2_.

There were two characteristic peaks in the Raman spectrum of MoS_2_, the layer number of MoS_2_ sample could be measured by the Raman spectrum between the E^1^_2g_ mode and the A_1g_ mode. It could be found by observing Figure 2a that the distance Δ*k* between the E^1^_2g_ characteristic peak and A_1g_ characteristic peak was 18 ± 0.1 cm^−1^, and the ratio of A_1g_/E^1^_2g_ was about 1.043, which indicates that the MoS_2_ sample was a monolayer [21]. Figure 2b shows the photoluminescence spectrum of the monolayer MoS_2_ sample at different points. The photoluminescence spectrum of monolayer MoS_2_ on SiO_2_/Si substrate had I and B exciton peaks, the strongest I exciton peak position of monolayer MoS_2_ was at 684.6 ± 0.5 nm, which could be explained by the direct exciton excitation [22]. The corresponding electron volt was at 1.82 ± 0.02 eV through the conversion relationship between wavelength and electron volts, which was consistent with the direct band gap width of monolayer MoS_2_. In addition, there was also the B exciton peak at 1.97 ± 0.02 eV due to the 3d orbital electron interaction of Mo atoms, which could further prove that the sample was the large-area high-quality monolayer MoS_2_.

In Figure 2c, the Raman spectrum intensity of monolayer MoS_2_ increased with the laser power increase. There was a blue shift of the E^1^_2g_ characteristic peak when the laser power increased, and the A_1g_ characteristic peak position did not change. This was because the MoS_2_ on the SiO_2_/Si substrate was an n-type doped semiconductor material [23]. Figure 2d shows the power photoluminescence spectrum of monolayer MoS_2_. The peak intensity of the photoluminescence spectrum increased when the laser power increased. At the same time, the relative positions between the I exciton peak and B exciton peak were red-shifted to some extent when the laser power increased. The reason was that the MoS_2_ on SiO_2_/Si substrate was the *n*-type doped material [24]. It is known from the above Raman spectrum and photoluminescence spectrum that the MoS_2_ sample was a monolayer.

## 3. The Discussion of Electrical Performance Results

The field effect transistor is the most basic electronic component in the digital logic circuits, which consists of the channel, a source electrode, a drain electrode, and the gate dielectric layer [25]. It can control the channel internal carrier density and the source–drain current by adjusting the gate voltage, which can achieve the current amplification and power amplification.

### 3.1. The Fabrication Process of Monolayer MoS_2_-Based FET

**Figure 3:**
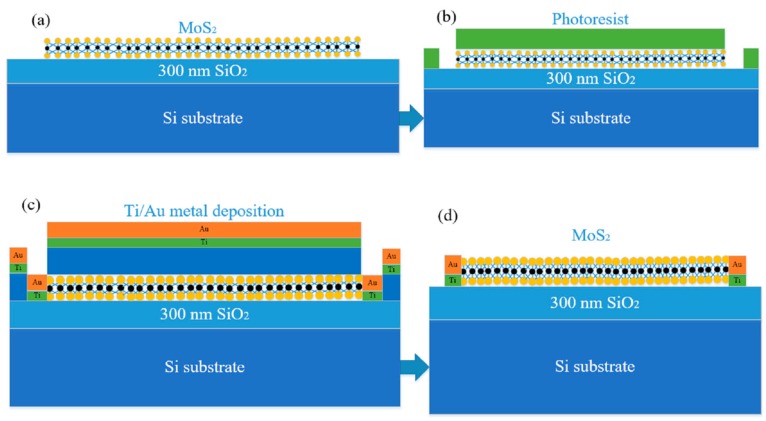
The main preparation process schematic diagram of the monolayer MoS_2_-based field effect transistor (FET; (**a**)) the growth process of the high-quality triangular monolayer MoS_2_; (**b**) the photolithography and the Ar plasma etching process; (**c**) the electron beam evaporation process; and (**d**) the cleaning process of the photoresist.

The monolayer MoS_2_-based back-gate FET can be fabricated by the photolithography, electron beam evaporation, and lift-off micromachining processes, the above steps used the polymethyl methacrylate (PMMA) resist process. The following is the specific process flow schematic diagram of monolayer MoS_2_-based FET [26]. First, the high-quality triangular monolayer MoS_2_ is grown on the surface of Si/SiO_2_ substrate by APCVD, as shown in Figure 3a; in Figure 3b, the pre-baking, gluing, exposing, and developing processes were performed to complete the photolithography, then using the photoresist as a mask, and the Ar plasma etching process was performed to remove the exposed MoS_2_ film, the photoresist was removed by the acetone solution, the ethanol solution was cleaned to determine the source and drain windows of monolayer MoS_2_-based FET; Subsequently, the 20 nm Ni/70 nm Au were used as the source-drain metal contact by the electron beam evaporation, as shown in Figure 3c; Next, the photoresist was dissolved in the acetone solution, and the metal solution was removed by the ethanol solution, as shown in Figure 3d.

Finally, the device was annealed in the vacuum environment of 180 °C for 2 h to remove the photoresist residue and decrease the contact resistance between the Ti/Au metal and monolayer MoS_2_. The fabrication of monolayer MoS_2_-based FET was completed, and the electrical performance of monolayer MoS_2_-based FET was analyzed and tested by the multi-function probe station and the B1500A semiconductor parameter analyzer (Santa Clara, CA, USA).

**Figure 4:**
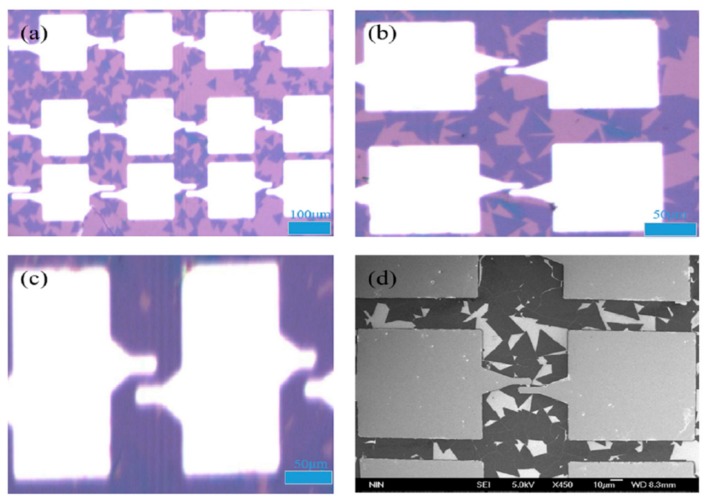
(**a**) The 10× objective optical microscope image; (**b**) the 50× objective optical microscope image; (**c**) the 100× objective optical microscope image of the monolayer MoS_2_-based back-gate FET; and (**d**) the FESEM image of the monolayer MoS_2_-based back-gate FET.

Figure 4a–c respectively show the 10×, 50×, and 100× objective optical microscope images of the monolayer MoS_2_-based back-gate FET, and the pink and blue patches in the optical images respectively represents the monolayer MoS_2_ on SiO_2_/Si substrate and the scale, it can be found that the area of monolayer MoS_2_ was usually 10–30 μm, and the size of the triangular monolayer MoS_2_ was relatively smaller compared to the metal electrodes, so the source and drain metal electrodes were divided into an electrode lead and the pad. The electrode lead (line width of 5 μm) was directly in contact with monolayer MoS_2_, and the pad of the source and drain metal electrodes was a square of 100 μm × 100 μm, which was for the electrical performance tests on the multi-function probe station [27]. The FESEM image of the monolayer MoS_2_-based back-gate FET is given in Figure 4d, it can be seen that the uneven brightness and blurred focus appeared on the surface of monolayer MoS_2_, which was affected by the charging effect of the SiO_2_/Si substrate during the FESEM scanning.

### 3.2. The Electrical Properties of Monolayer MoS_2_-Based FET

**Figure 5:**
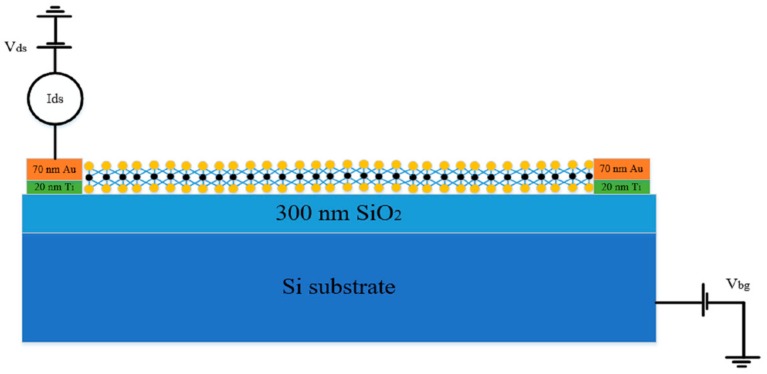
The electrical test structure of the monolayer MoS_2_-based back-gate FET.

Figure 5 shows the electrical test structure schematic diagram of the monolayer MoS_2_-based back-gate FET. The gate oxide of the back gate electrode was 300 nm SiO_2_, and monolayer MoS_2_ was used as the conductive channel material. To decrease the contact resistance, the source/drain metal electrodes were made of 20 nm Ti/70 nm Au. The 20 nm Ti metal acted as the adhesion layer between monolayer MoS_2_ and the Au, which could prevent the Au metal falling off from the MoS_2_ film [28]. Besides, the Ti metal could also facilitate the formation of the ohmic contact between the monolayer MoS_2_ sample and the Au electrode.

**Figure 6:**
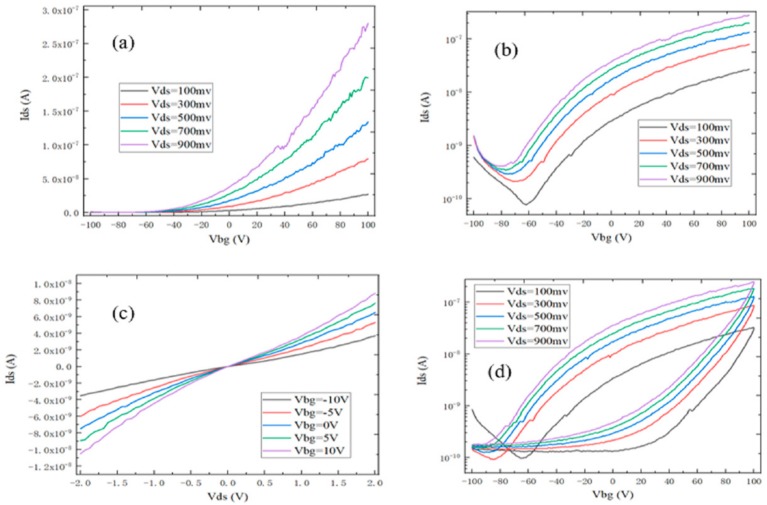
The monolayer MoS_2_-based back-gate FET. (**a**) The I_ds_–V_bg_ transfer curve; (**b**) the I_ds_–V_bg_ transfer curve with the ordinate semi-logarithmic coordinate; (**c**) the I_ds_–V_ds_ output curve; and (**d**) the hysteresis loop of MoS_2_ FET under different V_ds_.

The transfer and output characteristics of monolayer MoS_2_-based back-gate FET are shown in Figure 6. It can be seen by observing Figure 6a that the on-state current of the FET increased with the source–drain voltage increase. When the source–drain voltage was 0.9 V, the on-state current was about 2.75 × 10^−7^ A/μm, which was far from the application requirement of the high-performance FET [29]. In Figure 6b, the gate voltage could effectively regulate the channel resistance and the source-drain current, which exhibited the better switching characteristics. The source–drain current decreased when the back-gate electrode was at the negative voltage, whereas the source–drain current increased when a forward voltage was applied to the back-gate electrode, so the monolayer MoS_2_-based FET was the *n*-type transmission, and the switching ratio could reach 10^3^. The reason was that monolayer MoS_2_ had the smaller grain size and the relatively poor quality, which had a major effect on the device performance.

Based on the *I_ds_*–*V_gs_* transfer curve, the field effect mobility can be calculated by using the following equation [30]:

(1) $$\mu =\frac{dI_{ds}}{dV_{gs}}\frac{L}{W\left(\varepsilon_{0}\varepsilon_{r}/d\right)V_{ds}}$$

The channel length of device *L* = 10 μm, the channel width of device *W* = 10 μm, the source and drain voltage *V_ds_* = 0.9 V, vacuum dielectric constant *ε*_0_ = 8.85 × 10^−12^ F/m, the relative dielectric constant of SiO_2_ *ε_r_* = 3.9, the thickness of SiO_2_ *d* = 300 nm, *dI_ds_*/*dV_gs_* is the slope of the transfer curve. Therefore, when the *V_ds_* is 0.9 V, the field effect mobility is approximately 0.86 ± 0.05 cm^2^/Vs according to the slope of the linear region between 40 V and 100 V. This is because the lattice structure of MoS_2_ grown by APCVD is not complete. There are the lattice defects, which can deteriorate the mobility of monolayer MoS_2_-based FET. Due to the large forbidden band width of monolayer MoS_2_, the lowest gate leakage current of MoS_2_ FET is at the 10^−12^ A when the gate voltage gradually increase from −100 to 100 V, so it is suitable for the low-power logic circuits. The lower gate leakage current can effectively decrease the leakage power, which can help to improve the lifetime of the device [31]. As shown in Figure 6c, the output curve of monolayer MoS_2_ FET was linear, the gate voltage could well control the output current, and the output current increased with the gate voltage increase, which indicates that the monolayer MoS_2_-based FET was an *n*-type carrier transmission. For the monolayer MoS_2_-based back-gate FET, the current was proportional to *V_ds_* in the linear regime at the small source-drain voltage, and the *I_ds_*–*V_ds_* curve of FET device exhibited the odd function characteristic with the good linearity and central symmetry when the *V_ds_* increased from −2 to 2 V. Moreover, the *V_gs_* had a significant regulation effect on the slope of the output curve, which indicates that the monolayer MoS_2_-based back gate FET could form the good ohmic contact between the Ti/Au metal and MoS_2_ channels. It can be found from Figure 6d that the transfer characteristic curve had the obvious hysteresis phenomenon. This is due to the fact that the channel material used monolayer MoS_2_, which was very sensitive to the environmental change [32]. The monolayer MoS_2_-based FET could absorb the moisture and impurity gases from the air, which would have the important impact on the electrical performance of the monolayer MoS_2_-based FET.

## 4. Conclusions

In this paper, the monolayer MoS_2_ on SiO_2_/Si substrate was grown by APCVD, and the MoS_2_ sample was characterized by the high resolution microscope, the Raman spectroscopy, photoluminescence spectroscopy, and field emission scanning electron microscopy, which could prove the existence of monolayer MoS_2_. In order to evaluate the quality of monolayer MoS_2_ systematically, the monolayer MoS_2_ was used as the channel material of the FET, and the back gate FET was fabricated on the monolayer MoS_2_. It could be found from the electrical parameters of FET that the ohmic contact could be formed between monolayer MoS_2_ and Ti/Au metal electrode, the gate leakage current and static power consumption were lower. At the same time, the on-state current was about 2.75 × 10^−7^ A/μm when the source-drain voltage was 0.9 V, both the switching ratio and the mobility increased to some extent, which still need further improvement. The growth process of MoS_2_ was optimized to obtain the higher quality monolayer MoS_2_, so that the monolayer MoS_2_-based FET could be applied to the future low-power optoelectronic integrated circuits.
